# Evolutionary relationships between heme-binding ferredoxin α + β barrels

**DOI:** 10.1186/s12859-016-1033-6

**Published:** 2016-04-18

**Authors:** Giriraj Acharya, Gurmeet Kaur, Srikrishna Subramanian

**Affiliations:** CSIR-Institute of Microbial Technology (IMTECH), Sector 39-A, Chandigarh, India

**Keywords:** Hemoprotein, Barrel packing, Heme, Iron metabolism, Protein evolution

## Abstract

**Background:**

The α + β barrel superfamily of the ferredoxin-like fold consists of a functionally diverse group of evolutionarily related proteins. The barrel architecture of these proteins is formed by either homo-/hetero-dimerization or duplication and fusion of ferredoxin-like domains. Several members of this superfamily bind heme in order to carry out their functions.

**Results:**

We analyze the heme-binding sites in these proteins as well as their barrel topologies. Our comparative structural analysis of these heme-binding barrels reveals two distinct modes of packing of the ferredoxin-like domains to constitute the α + β barrel, which is typified by the Type-1/IsdG-like and Type-2/OxdA-like proteins, respectively. We examine the heme-binding pockets and explore the versatility of the α + β barrels ability to accommodate heme or heme-related moieties, such as siroheme, in at least three different sites, namely, the mode seen in IsdG/OxdA, Cld/DyP/EfeB/HemQ and siroheme decarboxylase barrels.

**Conclusions:**

Our study offers insights into the plausible evolutionary relationships between the two distinct barrel packing topologies and relate the observed heme-binding sites to these topologies.

**Electronic supplementary material:**

The online version of this article (doi:10.1186/s12859-016-1033-6) contains supplementary material, which is available to authorized users.

## Background

The “dimeric α + β barrel superfamily” (Pfam CL0032, SCOP identifier 54909) is an evolutionarily conserved group of protein families found in all three kingdoms of life [1–4]. Proteins of this superfamily include the antibiotic biosynthesis monooxygenases (ABM; PF03992) [5], transcription regulators of the AsnC family (PF01037) [6], polyketide synthases (PF04673) [7], ether compound degraders (PF07110) [8], etc. Some families of this superfamily are involved in degradation and synthesis of heme (Iron-regulated surface determinants, IsdG and IsdI, which are members of the ABM family and HemQ, respectively) [9, 10]. This superfamily is also referred to as the CDE superfamily in literature, CDE being an acronym for the chlorite dismutase (Cld, PF06778), dye-decolorizing peroxidase (DyP, PF04261) and EfeB heme-binding families [2, 11].

**Fig. 1:**
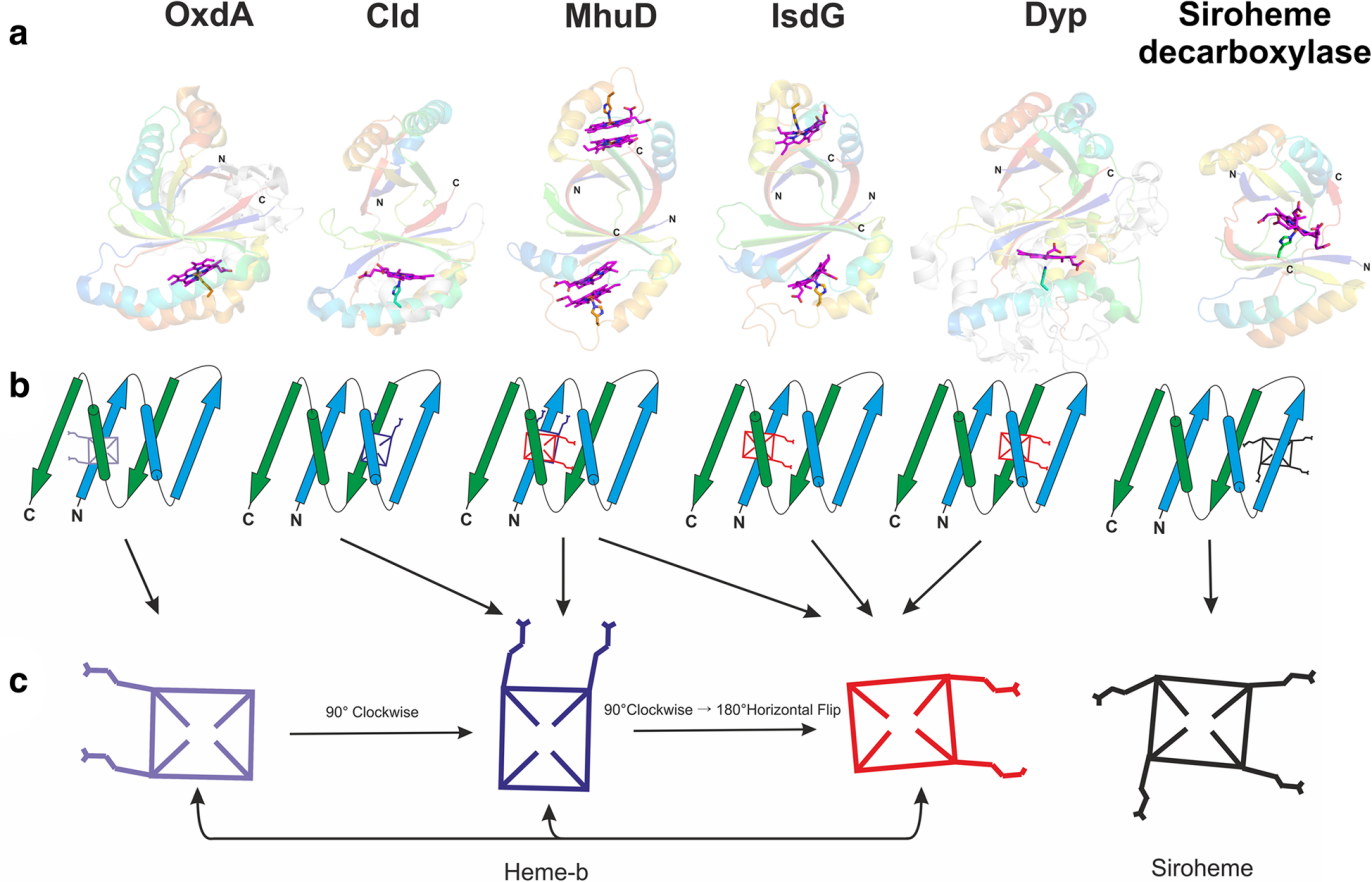
Representative structures of the heme-binding ferredoxin α + β barrel superfamily. (**a**) Ribbon diagram of the structures of OxdA (PDB identifier 3A16), Cld (PDB identifier 3NN1), MhuD (PDB identifier 3HX9), IsdG (PDB identifier 2ZDP), DyP (PDB identifier 3VXJ) and Siroheme decarboxylase (PDB identifier 4UN1). The individual ferredoxin-like folds in all structures have been colored from their N- to C-termini in chainbow coloring scheme of PyMOL and are faded to highlight the position of heme (magenta). Elements that do not constitute a part of the core of the ferredoxin-like fold are colored white. (**b**) A comparison of heme binding sites using topology diagrams. (**c**) Orientation of heme-moieties in different proteins of the ferredoxin α + β barrel superfamily. Here, the porphyrin ring of heme and siroheme substituents is shown as a rectangle and the extensions from the rectangle denote the propionate side chains

Heme, a metal-porphyrin compound, is associated with at least six different families of this superfamily [2, 10, 12, 13]. Of these, three families, namely, Cld, DyP and aldoxime dehydratase (OxdA, PF13816) have been identified to bind heme as a cofactor [12–14], while IsdG and HemQ bind heme as a substrate and product, respectively [9, 10] (Fig. 1a). For members of the EfeB family, heme has been proposed to play a role in the assimilation of iron [15] and in the extra-cytoplasmic transport of the protein [16]. Heme is seen to bind at two spatially distinct regions in the barrel [2]. IsdG [17], HmoB [18], MhuD [19] and OxdA [14] share similar spatial location of the heme-binding site [2], while Cld [20], DyP [21] and EfeB [22] bind heme at a different location [2]. The exact location of the heme-binding histidine and orientation of the heme moiety vary even among proteins with the similar spatial location of heme [2] (Fig. 1b, c). In addition to these heme-binding families, a recently structurally characterized protein, siroheme decarboxylase, which is a member of the AsnC transcription regulator family, is known to function in the alternative heme biosynthesis pathway in some bacteria and archaea [23, 24]. Siroheme decarboxylase has been shown to bind siroheme, a metabolic intermediate in the alternative heme synthesis pathway, and convert it to didecarboxysiroheme [23–25] (Fig. 1).

The proteins of this superfamily are made up of two domains that pack together as a closed barrel (SCOP identifier 54909) (Fig. 1a). Each domain has a ferredoxin-like fold (SCOP identifier 54861) with two repeats of a β-α-β structural motif that form a two-layer α + β sandwich (β1-α1-β2-β3-α2-β4) with an antiparallel β-sheet. The antiparallel β-sheet from two such ferredoxin-like domains interact and form a barrel with an all β layer on the inside decorated with α-helices on the outside (Fig. 1a). The two ferredoxin-like domains involved in barrel formation may be present as tandemly duplicated and fused domains on a single polypeptide chain or as standalone domains from separate protein chains. Based on the initially determined structures, this superfamily was named the “dimeric alpha + beta barrel [54909]” by SCOP (v1.63) as two ferredoxin-like domains were seen to dimerize to form the barrel and this terminology has been used extensively in the literature. However, this nomenclature is a misnomer as many structures with two ferredoxin-like domains from a single polypeptide that adopts a barrel architecture are now available. Thus, in order to avoid confusion, we refer to this superfamily as the ferredoxin α + β barrel and suggest the adoption of this nomenclature for this superfamily. Further, the ferredoxin-like domains from different chains may form a barrel by combining with identical domains (homodimers) or evolutionarily related domains (heterodimers). For example, in most IsdG homologs a single ferredoxin-like domain is present per protein chain which homodimerizes to form the barrel [17, 26, 27] (Fig. 1), while in most members of the Cld, Dyp, EfeB, HemQ and OxdA families, two duplicated and fused ferredoxin-like domains are present in tandem on the same polypeptide to form the barrel [12, 14, 22, 28, 29]. In several of the ferredoxin α + β barrel families, biologically significant higher oligomeric forms are observed [2, 12, 14, 28, 29]. For example, proteins of the DyP, Cld, EfeB, HemQ and OxdA families are known to contain dimeric, trimeric, pentameric and hexameric copies of the ferredoxin α + β barrel [12, 14, 28, 29], and proteins of the muconolactone isomerase family and sulphur oxygenase reductase are known to exist as homo decamer and an oligomer made of 24 barrel units, respectively [30, 31].

While many studies describing the structural and biochemical characterization of these proteins are available, an evolutionary basis for the emergence of different heme-binding sites in the ferredoxin α + β barrel and rationale for the apparently different barrel packing is not available. Here, we present a comparative view of the different heme-binding ferredoxin α + β barrel families based on evolutionary considerations and identify two different barrel packing modes of the ferredoxin-like domains. The evolutionary scenarios that likely resulted in the topologically different packing modes are discussed. Further, we provide a comparative topological- and structural view of the various heme-binding sites in the versatile ferredoxin α + β barrel. It is particularly interesting to observe how the ferredoxin-like protein domain has evolved to utilize different interfaces for performing varied functions around the heme moiety. This work could be potentially useful for future protein structural and evolutionary studies and in understanding the spatial packing of protein domains.

## Results and discussion

The structure of the ferredoxin α + β barrel superfamily contains two ferredoxin-like domains that may be present on a single polypeptide either as tandemly duplicated and fused domains or as independent domains on two different polypeptides. Further, the barrel may involve packing of identical domains i.e. same gene products (homodimerization) or non-identical domains i.e. duplicated but not fused gene products (heterodimerization) or of tandemly duplicated and fused domains. The arrangement of the ferredoxin-like domains in the heme-binding families of this α + β barrel superfamily is summarized in Table 1. Most IsdG-like proteins form barrels via homodimerization of the ferredoxin-like domains. In contrast, proteins of the Cld, DyP, EfeB, HemQ and OxdA families have a α + β barrel made up of tandemly duplicated and fused ferredoxin-like domains on a single polypeptide. Similar to these families, the HmoB (PDB identifier 4OZ5), an IsdG homolog, is seen to possess tandemly duplicated ferredoxin-like domains which pack into a barrel. Structures of siroheme decarboxylase from two organisms, *Hydrogenobacter thermophilus TK-6* and *Desulfovibrio desulfuricans* have been determined recently [23, 24]. The barrels of siroheme decarboxylase display both aforementioned arrangements, i.e. two contiguous duplicated and fused ferredoxin-like domains on a single protein chain in the *H. thermophilus TK-6* siroheme decarboxylase (PDB identifier 4CZC) and two ferredoxin-like domains from different chains in *D. desulfuricans* siroheme decarboxylase (PDB identifier 4UN1). However, in the *D. desulfuricans* siroheme decarboxylase the monomeric ferredoxin-like domains are products of evolutionarily related genes and thus, the barrel is a result of heterodimerization of ferredoxin-like domains, unlike the homodimeric IsdG barrel.

**Table 1:** Barrel packing topologies observed in heme-binding members of the ferredoxin α + β barrel superfamily

Arrangement of ferredoxin-like domains in the barrels	Heme-binding ferredoxin α + β barrel family representatives	Barrel topology	β-strand of the ferredoxin-like domain involved in barrel packing
Homo-dimerization	IsdG and IsdI-like (PDB identifiers 2ZDO, 2ZDP, 3LGM, 3LGN, 3QGP, 4FNH, 4FNI)	Type-1	β2---β4; β4---β2
	MhuD (PDB identifiers 3HX9, 4NL5)		
Hetero-dimerization	Siroheme decarboxylase (PDB identifier 4UN1)		
Dimerization of tandemly duplicated and fused domains	HmoB (PDB identifiers 4FVC, 4JOU, 4OZ5), Chlorite dismutase (PDB identifiers 2VXH, 3NN1, 3NN2, 3NN3, 3NN4, 3Q08, 3Q09, 3QPI, 4 M05, 4 M06, 4 M07, 4 M08, 4 M09), Dyp-type peroxidase (PDB identifier 2D3Q, 2IIZ 3AFV, 3MM1, 3MM2, 3MM3, 3QNR, 3QNS, 3VEC, 3VED, 3VEF, 3VEG, 3VXI, 3VXJ, 4 AU9, 4G2C, 4GRC, 4GS1, 4GT2, 4GU7, 4HOV, 4UZI, 4W7J, 4W7K, 4W7L, 4W7M, 4W7N, 4W7O), EfeB (PDB identifiers 2Y4E, 2Y4F, 3O72), Siroheme decarboxylase (PDB identifier 4CZC)		
	Aldoxime dehydratase (PDB identifiers 3A15, 3A16, 3A17, 3A18, 3 W08)	Type-2	β2---β2; β4---β4

**Fig. 2:**
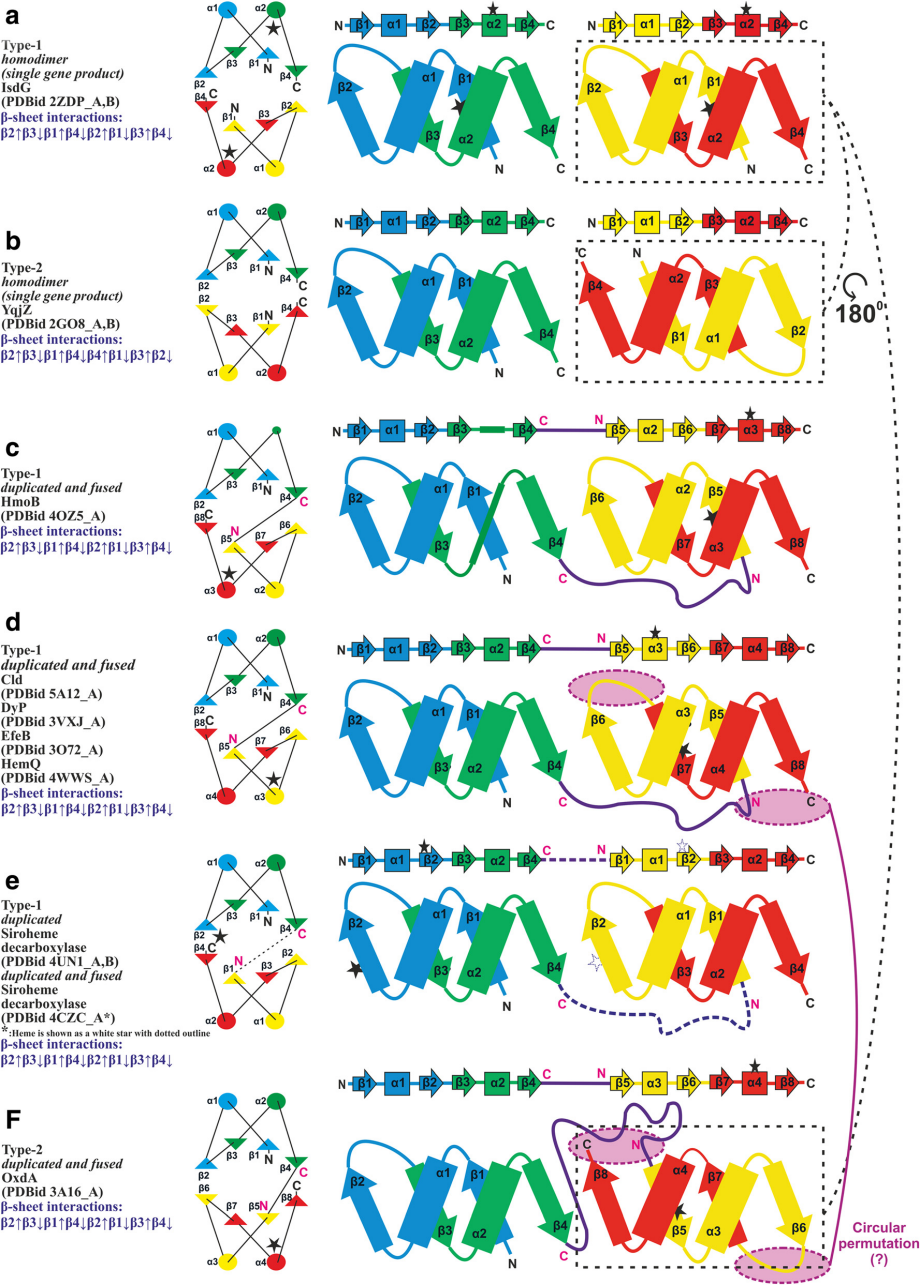
A comparative view of the packing topologies of the ferredoxin α + β barrels. (**a-f**) Left-side diagrams show a top view of the barrels with β-strands indicated as triangles and α-helices as circles. Right-side diagrams show an opened-up view of the barrel with two ferredoxin-like domains placed side-by-side to show β-strand interactions. Only one pair of interacting β-strands can be seen as the other pair is formed by the peripheral-β-strands. The linear arrangement of the secondary structure elements is shown above the opened-up view, with β-strands indicated as *arrows* and α-helices as *rectangles*. Each barrel is constituted of four β-α-β units from two ferredoxin-like domains, colored *blue*, *green*, *yellow* and *red*. Heme is shown as a *black star* in all but *H. thermophilus* siroheme decarboxylase where it is shown as a *white dotted*-outlined star. N- and C-termini of the protein chains are labeled ‘N’ and ‘C’ in *black* while those of individual ferredoxin-like domains in barrels with duplicated domains are in *pink*

Though two ferredoxin-like domains pack to form a structurally similar α + β barrel in all families, we identify two non-identical packing modes of these domains based on the orientation of the two ferredoxin-like domains with respect to each other within the barrel, which we refer to as Type-1 and Type-2 (Table 1, Fig. 2). This orientation determines which β-strands from the adjacent domains are involved in forming hydrogen bonds to constitute the barrel (Table 1, Fig. 2). In Type-1 packing, the two ferredoxin-like domains are oriented such that the N- and C-termini of both the domains are present on one end of the barrel, while in Type-2, the two ferredoxin-like domains are oriented such that the N- and C-termini of one of them are present along one end of the barrel and the N- and C-termini of the other domain are present on the opposite end (Fig. 2a). Such a packing results in an antiparallel β-strand interaction between the β2 from one ferredoxin-like domain and β4 from the other domain in the Type-1-packing mode (β2↑ β3↓ β1↑ β4↓ β2↑ β3↓ β1↑ β4↓), whereas in the Type-2-packing mode, the β2 strands from each of the ferredoxin-like domains interact with the other (β2↑ β3↓ β1↑ β4↓ β4↑ β1↓ β3↑ β2↓) (Fig. 2f). Type-1-packing mode typified by IsdG-like proteins is observed in a majority of the heme-binding proteins of the ferredoxin α + β barrel superfamily, *viz*., members of the ABM and Cld/DyP/EfeB/HemQ families. Type-2-packing is mostly confined to the OxdA family members and a group of non-heme-binding ferredoxin α + β barrel proteins typified by the hypothetical protein YqjZ (PDB identifier 2GO8) (Fig. 2b). Interestingly, YqjZ-like proteins (PDB identifiers 2GO8, 2FB0, 4NPO) are members of IsdG-ABM family (PF03992) but with a barrel packing resembling that of OxdA. Thus, we observe two different packing modes even between closely related proteins of the ABM ferredoxin α + β barrel family.

**Fig. 3:**
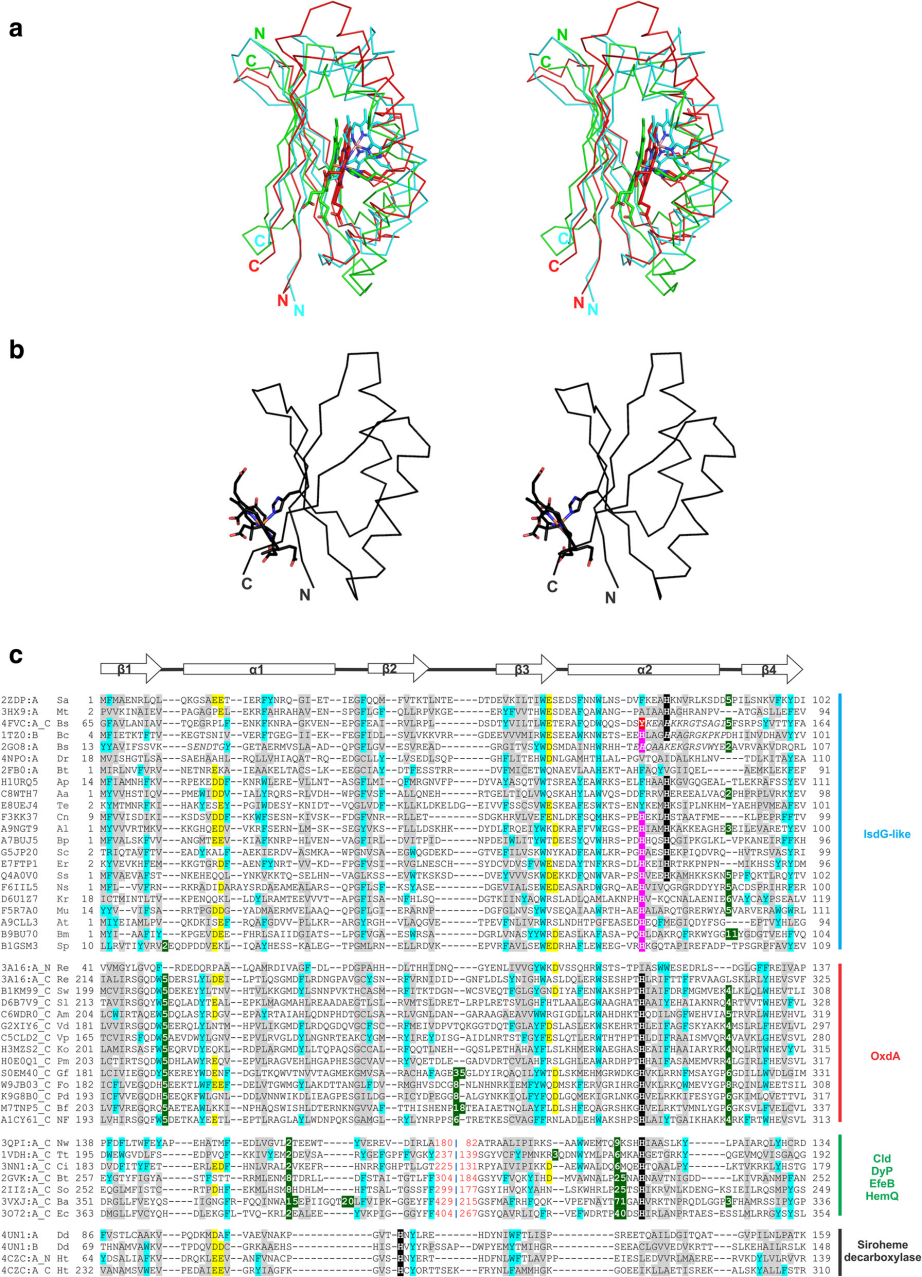
Structural and sequence features of the heme-binding region in IsdG-like, OxdA, Cld/Dyp/EfeB/HemQ, and siroheme decarboxylase proteins. (**a**) Structure superimposition stereo diagram of the C_α_ backbone of IsdG-like ferredoxin-like monomer (PDB identifier 2ZDP; cyan), the C-terminal domain of OxdA-like (PDB identifier 3A16_A; *red*) and the C-terminal domain of Cld/DyP/EfeB/HemQ-like (PDB identifier 3NN1_A; *green*). (**b**) Stereo diagram of a ferredoxin-like domain of siroheme decarboxylase (PDB identifier 4UN1_A). In panels (**a, b**), heme and side chain of the heme-binding histidine is shown in stick form. **c** Structure-based MSA of IsdG, OxdA, Cld/Dyp/EfeB/HemQ and siroheme decarboxylase. PDB identifier/UniProt ID, organism name, start, and end for each sequence are mentioned. The region of circular permutation is indicated by ‘|’ and the sequence number around this region is colored *red*. The secondary structure diagram for a ferredoxin-like domain is indicated above the alignment, where arrows represent β-strands and rectangles indicate α-helices. The number of omitted residues for large insertions is boxed in *green*. The sequence region(s) corresponding to disorder in structure or structurally not superimposable regions is italicized. Organism abbreviations: Sa-*Staphylococcus aureus*, Mt-*Mycobacterium tuberculosis*, Bs-*Bacillus subtilis*, Bc-*Bacillus cereus*, Dr-*Deinococcus radiodurans*, Bt-*Bacteroides thetaiotaomicron*, Ap-*Acetobacter pasteurianus*, Aa-*Alicyclobacillus acidocaldarius*, Te-*Taylorella equigenitalis*, Cn-*Candidatus nitrosoarchaeum*, Al-*Acholeplasma laidlawii*, Bp-*Beggiatoa sp*., Sc-*Streptococcus criceti*, Er-*Erysipelothrix rhusiopathiae*, Ss-*Staphylococcus saprophyticus*, Ns-*Novosphingobium sp*., Kr-*Ktedonobacter racemifer*, Mu-*Methyloversatilis universalis*, At-*Agrobacterium tumefaciens*, Bm-*Burkholderia multivorans*, Sp-*Streptomyces sp*., Re-*Rhodococcus erythropolis*, Sw-*Shewanella woodyi*, Sl-*Streptomyces albus*, Am-*Actinosynnema mirum*, Vd-*Verticillium dahliae*, Vp-*Variovorax paradoxus*, Ko-*Klebsiella oxytoca*, Pm-*Patulibacter medicamentivorans*, Gf-*Gibberella fujikuroi*, Fo-*Fusarium oxysporum*, Pd-*Penicillium digitatum*, Bf-*Botryotinia fuckeliana*, Nf-*Neosartorya fischeri*, Nw-*Nitrobacter winogradskyi*, Tt-*Thermus thermophilus*, Ci-*Candidatus Nitrospira*, So-*Shewanella oneidensis*, Ba-*Bjerkandera adusta*, Ec-*Escherichia coli*, Dd-*Desulfovibrio desulfuricans*, Ht-*Hydrogenobacter thermophilus*

Although non-identical, the two barrel packing modes may be rationalized by rotating one of the ferredoxin-like domains in Type-1 proteins by 180^0^, which would result in a Type-2 packing (Fig. 2). The ferredoxin-like domain possesses internal symmetry, which allows us to superimpose two such domains even after rotating them with respect to each other, as in either case, i.e. with or without rotation, we align similarly arranged symmetric β-α-β repeats. The rotation of one of the domains of IsdG with respect to OxdA further allows us to rationalize the different locations of the heme-binding-histidine in IsdG and OxdA-like proteins (Fig. 2). Studies have revealed the presence of two heme-binding active sites, one in the cleft of each ferredoxin-like domain of the barrel in IsdG and IsdI [32]. The heme-degrading catalytic triad is made up of Asn6, Trp66, and His76 residues, where the Trp residue is implicated in ruffling of the heme moiety [32, 33]. In the structure of OxdA from *Rhodococcus sp.*, which has two ferredoxin-like domains joined by a linker region and additional secondary structural elements, a single heme-binding site is present in the cleft of the C-terminal ferredoxin-like domain of the barrel [14]. The apo- and holo-structures of OxdA reveal His299 as the proximal residue for heme and two functionally-important distal residues, Ser219 and His320 [14], which help in orienting the heme-bound substrate for the catalytic elimination of OH group of aldoxime [34, 35]. A comparison of the heme-binding domains of IsdG and OxdA reveals significant sequence as well as structure similarity around the heme-binding site (Fig. 3, Additional file 1: Figure S1). For a detailed comparison of protein-heme interactions, the readers may refer to Celis and DuBois, 2015 [2]. Heme is bound at a similar spatial location in IsdG and OxdA barrels [2] and the heme-binding histidine is contributed by the α2 of the individual ferredoxin-like domains [14, 32]. However, the histidine contributing α-helix is not topologically equivalent (Fig. 2), as in OxdA, heme is bound on α4 of the barrel which is topologically equivalent to the α1, and not the heme-binding α2, of the ferredoxin-like domain in the IsdG barrel (Table 2, Fig. 2a). Thus, an 180^0^ rotation of the C-terminal domain of IsdG not only helps rationalize the barrel packing-modes but also helps correctly superimpose the heme-binding α-helices of IsdG and OxdA.

**Table 2:** Detailed comparison of different heme-/siroheme-binding ferredoxin α + β barrel families

	IsdG homologs	OxdA	Cld/DyP/EfeB/HemQ	Siroheme decarboxylase
Number of heme/siroheme per barrel	2 (IsdG/IsdI, PDB identifier 2ZDO/2ZDP) (1/monomer)	1 (in C-terminal ferredoxin-like domain)		1 (between the two ferredoxin-like domains)
	4 (MhuD, PDB identifier 3HX9) (2/monomer)			
	1 (HmoB, PDB identifier 4OZ5) (1 in C-terminal ferredoxin-like domain)			
Heme/siroheme binding pocket	Cleft of the ferredoxin-like domain			Barrel cavity
Function of heme/siroheme	Substrate	Cofactor	Cofactor, Product (HemQ)	Metabolic intermediate
Shape of heme/siroheme	Ruffled	Ruffled	Planar	Ruffled siroheme
Location of the proximal His-axial residue	α2 of ferredoxin-like domain (α3 in the contiguous duplicated domains in HmoB)	α2 of the ferredoxin-like domain (α4 in the contiguous duplicated domains). These are spatially not equivalent to α2 of IsdG homologs.	α1 of the ferredoxin-like domain (α3 in the contiguous duplicated domains). These are spatially equivalent to OxdA.	β2 of the ferredoxin-like domain of chain A in *D. desulfuricans. * β6 of the contiguous duplicated domains of the barrel in *H. thermophilus*
Distal heme residue	Asparagine	Histidine	Arginine	Histidine

The second heme-binding site, structurally distinct from IsdG and OxdA [2], is seen in Cld/DyP/EfeB/HemQ proteins, where the heme-binding histidine though contributed by the C-terminal domain, is located on α3 of the barrel. Further, α3 of Cld/DyP/EfeB/HemQ barrels is also topologically equivalent to the α1 of the ferredoxin-like domain of IsdG, similar to the OxdA proteins (Table 2, Fig. 2d, f), which allows us to relate the C-terminal domains of OxdA and Cld/DyP/EfeB/HemQ barrels by a circular permutation. Hence, by considering, first, a 180^0^ rotation of the C-terminal domain in OxdA with respect to the domain of IsdG, and second, a circular permutation in the heme-binding domain of Cld/DyP/EfeB/HemQ proteins relative to that of OxdA, we were able to align the heme-binding site in the IsdG ferredoxin-like monomer (PDB identifier 2ZDP), and the C-terminal domains of the OxdA (PDB identifier 3A16_A) and the Cld/DyP/EfeB/HemQ (PDB identifier 3NN1_A) barrels (Fig. 3a). The IsdG ferredoxin-like monomer (PDB identifier 2ZDP) and 180^0^-rotated C-terminal domain of OxdA (PDB identifier 3A16) could be manual superimposed with an RMSD of 1.16 Å over 50 pairs of C_α_-atoms, while the C-terminal domain of OxdA (PDB identifier 3A16) and Cld (PDB identifier 3NN1) could be superimposed with an RMSD of 2.63 Å over 53 pairs of C_α_-atoms.

Homologs of IsdG, OxdA, and Cld/DyP/EfeB/HemQ were obtained by sequence similarity searches initiated using JackHMMER [36]. A multiple sequence alignment (MSA) of IsdG, the C-terminal domains of OxdA and the C-terminal domains of Cld/DyP/EfeB/HemQ members is presented in Fig. 3c. As shown in the MSA, the exact location of the heme-binding histidine (highlighted black in Fig. 3c) is variable among members of the ferredoxin α + β barrel proteins. MSA of the structurally characterized IsdG and OxdA proteins reveal that the histidine is present at non-identical positions in the ferredoxin-like domain, i.e. histidine in OxdA proteins is three residues away from the histidine in IsdG. Structurally, this implies that the heme-binding histidine in OxdA is one α-helical-turn before the histidine of IsdG (Fig. 3a). However, we could find several sequences of the IsdG family (UniProt ID: F6IIL5, D6U1Z7 in Fig. 3c) which also possess a histidine at an equivalent position to OxdA (highlighted in magenta in Fig. 3c), suggesting a possible migration of heme-binding site in these evolutionarily related ferredoxin-like domains. In some of the IsdG proteins (UniProt ID: E7FTP1, G5JP20 in Fig. 3c), two histidines are present, one of which is at the position seen in IsdGs and the other at the position corresponding to that of OxdA heme-binding histidine. Besides the conservation of the pattern of histidines, we observe conservation of several hydrophobic (highlighted in gray in Fig. 3c) and aromatic residues (highlighted in cyan in Fig. 3c) which is indicative of an evolutionary relation among the IsdG and OxdA proteins and consequently, their heme-binding sites. Further, automated sequence similarity searches using FFAS [37], initiated with the full-length OxdA proteins could find high-scoring matches to members of the ABM/IsdG family, but not to other members of the Pfam “dimeric α + β barrel” clan (CL0032). For example, OxdA (PDB identifier 3A16, residues 6–367) could find IsdI (PDB identifier 2ZDP_A, Score = −14.1), Antibiotic biosynthesis monooxygenase (PDB identifier 2RIL_A, Score = −13.6) and YqjZ (PDB identifier 2G08_A, Score = −13.5) as the top scoring matches in searches initiated against the PDB database. An FFAS score lower than −9.5 is associated with < 3 % false positive hits and higher confidence may be vested with hits with scores lower than this [37]. Statistically significant sequence similarity is used as a measure of homology [38] and these scores reveal a rather close relation between the OxdA and IsdG/ABM-family members as compared to other families of the ferredoxin α + β barrel.

A plausible evolutionary scenario that could have led to the emergence of different packing modes and heme-binding sites is detailed below. However, from a structural viewpoint as both the ferredoxin-like domain packing modes lead to formation of a similar barrel, thus, any apparent reason for one type being favored over the other during evolution is not evident. The simplest manner in which a α + β barrel could have emerged is by homodimerization of identical ferredoxin-like domains. The ferredoxin-like domains could have packed in two non-identical modes (similar to Type-1 in IsdG and Type-2 in YqjZ, PDB identifier 2ZDO and 2GO8, respectively). Further, duplication and divergence would have likely resulted in non-identical ferredoxin-like domains that could heterodimerize. The heterodimerization could also be of either type, but the structure for only the Type-1 packing mode is known currently (as seen in siroheme decarboxylase, PDB identifier 4UN1). Likewise, a tandem duplication and fusion of the ferredoxin-like domains would have resulted in a barrel formed from two domains present on a single polypeptide. However, these could again pack in the two aforementioned modes dictated possibly by the flexibility of the linker region between the domains (similar to Type-1 in HmoB and Type-2 in OxdA, PDB identifiers 4OZ5 and 3A16, respectively). The presence of tandemly duplicated ferredoxin-like domains is likely to have eased the evolutionary pressure for heme binding on both the domains and thus, only one domain retained the heme-binding histidine (mostly the C-terminal domain as seen in HmoB, Cld, DyP, EfeB, OxdA). The N-terminal domain, thereafter, likely served a mere structural role in barrel packing and is even seen to have lost some of the secondary structural elements of the ferredoxin α + β barrel scaffold, for example in the HmoB protein. The structure of HmoB (PDB identifiers 3TVZ, 4FVC, 4JOU, 4OZ5) reveals the degradation of the α-helix, equivalent to the heme-binding α-helix of the ferredoxin-like domain of evolutionary-related IsdG proteins, in its N-terminal domain (Fig. 2). Further, although the Cld/DyP/EfeB/HemQ barrels have a Type-1 packing similar to that seen in IsdG, their heme-binding histidine is on an α-helix which is topologically equivalent to that of OxdA Type-2-packed barrel (Fig. 2), thus allowing us to relate the C-terminal domains of OxdA and Cld/DyP/EfeB/HemQ by a probable circular permutation event. Nonetheless, the possibility of having acquired heme-binding ability independent of other ferredoxin α + β barrels cannot be ruled out. Interestingly, initial structural studies of the DyP family proteins attributed the presence of heme at two different positions [39-41], but subsequent studies established a single heme-binding site in DyP proteins based on conservation of one of the proximal histidine [13, 21]. Further, in another study concerning the structural characterization of HmoB from *B. subtilis* (PDB identifier 4FVC_A), heme is seen to bind to a nearby Tyr residue (boxed in red in Fig. 3c) when the secondary structure element with the proximal histidine is disordered. Intriguingly, the position of this Tyr residue is equivalent to the heme-binding histidine in OxdA, thus providing additional evidence in favor of evolutionary relatedness of IsdG and OxdA families and migration of the heme-binding site. Thus, these examples together with the analysis of heme-binding barrels reveal the versatility of the ferredoxin-like domain to accommodate heme at different locations.

A vivid example of the independent emergence of heme-like-moiety-binding is that of siroheme decarboxylase, an enzyme of the alternative heme synthesis pathway which catalyzes the conversion of siroheme to 12, 18 didecarboxysiroheme [23–25]. Evidence in favor of independent acquisition of siroheme-binding in siroheme decarboxylase include, firstly, that siroheme binds at a completely different region, i.e. inside the barrel cavity, as compared to other ferredoxin α + β barrel proteins which bind heme within the cleft of the ferredoxin-like domain (Figs. 1a and 3b, Table 2) and secondly, their probable homologous relation to proteins of the AsnC family [42] which are known to primarily function in transcription regulation pathways [2, 43]. The sequences of the ferredoxin-like domains of siroheme decarboxylase (PDB identifiers 4UN1, 4CZC) are not currently classified in Pfam but find high-scoring matches to AsnC family proteins using PSI-BLAST. For example, *H. thermophilus* siroheme decarboxylase (PDB identifier 4CZC_A) finds matches to uncharacterized HTH-type transcriptional regulator PH1519 (UniProt identifier O59188, with an E-value 3e-06) and uncharacterized HTH-type transcriptional regulator PYRAB06490 (UniProt identifier Q9V0Y9, with an E-value of 3e-05).

We refer to the mode of siroheme-binding in siroheme decarboxylase (PDB identifiers 4UN1, 4CZC) as the third mode of heme binding observed in ferredoxin α + β barrel families, as unlike the aforementioned families which bind heme in the cleft of the ferredoxin-like domain, siroheme decarboxylase binds siroheme in the barrel cavity formed by the two ferredoxin-like domains (Fig. 1a). The histidine which binds siroheme is located on a β-strand of the ferredoxin-like domain and not on a α-helix like the other heme-binding α + β barrels (Fig. 2e). Thus, this mode of heme binding cannot be related to the IsdG, Cld/DyP/EfeB/HemQ and OxdA by any of the evolutionary mechanisms discussed above, such as circular permutation, rotation of the ferredoxin-like domains, etc. Interestingly, though the two structurally characterized siroheme decarboxylases (PDB identifiers 4UN1, 4CZC) possess a similar barrel cavity as the site for siroheme-binding, the siroheme moiety is bound and oriented differently in these proteins. While the heme-binding histidine is located at the same position, i.e. on the β2 strand, the barrel reveals that the histidine is contributed by β2 of chain A in *D. desulfuricans* (PDB identifier 4UN1) and from β6 of the barrel of *H. thermophilus* (PDB identifier 4CZC). *H. thermophilus* siroheme decarboxylase (PDB identifier 4CZC) as mentioned above has duplicated and fused ferredoxin-like domain (single polypeptide) and thus, the β6 of *H. thermophilus* (PDB identifier 4CZC) is equivalent to β2 of an individual ferredoxin-like domain of *D. desulfuricans* (PDB identifier 4UN1). We observe that in each of these proteins, both the ferredoxin-like domains of the barrel possess a histidine residue on the β2 strand. A superimposition of the siroheme decarboxylase structures from *D. desulfuricans* and *H. thermophilus*, reveals that the bound siroheme molecules are located at spatially distinct locations and conformation changes to a few side-chains of the siroheme moiety could allow the ferredoxin α + β barrel to accommodate two siroheme molecules simultaneously, without steric clashes.

## Conclusions

We compared the various modes in which the ferredoxin-like domains may pack in the heme-binding ferredoxin α + β barrel families. We find that the ferredoxin-like domains may pack in two modes to form the barrel, which differ in the orientation of the constituent domains. Of the 18 families of ferredoxin α + β barrel superfamily (as classified by Pfam), Type-1 packing mode is seen in proteins belonging to 17 families of which proteins from the ABM, Cld, DyP and AsnC families are known bind heme and Type-2 is observed only in the heme-binding OxdA family proteins and the non-heme YqjZ-like proteins of the ABM family. Our analysis helps rationalize the underlying relationships between the different heme-binding sites and barrels of IsdG, OxdA and Cld/DyP/EfeB/HemQ proteins.

## Methods

### Dataset of ferredoxin α + β barrel superfamily proteins used in the present study

Representatives of the ferredoxin α + β barrel superfamily were selected from the SCOP and PDB databases after considering the following criteria. The presence of bound heme/heme-like ligand molecules to ferredoxin-like domain(s) was the primary criteria followed by a preference for structures without disordered regions and/or non-natural mutation(s) with maximum sequence length coverage. X-ray diffraction structures were preferred over NMR structures and the one with the highest resolution was selected. Representative structures that were selected included IsdG (PDB identifier 2ZDP_A), MhuD (3HX9_A), chlorite dismutase (PDB identifier 3NN1_A), DyP-type peroxidase (PDB identifier 3VXJ_A), EfeB (PDB identifier 3O72_A), HemQ (PDB identifier 1VDH_A) and aldoxime dehydratase (PDB identifier 3A16_A). Siroheme decarboxylase, which binds siroheme and whose structure was characterized recently, was also included in the representative dataset (PDB identifiers 4UN1_A, B, 4CZC_A) along with the representative of AsnC transcription regulator family (PDB identifier 2E7X_A). Other related proteins were obtained by sequence and structural similarity searches as outlined below (Additional file 2: Figure S2, Additional file 3).

### Sequence-based methods

The sequences of the selected representatives were used to initiate sequence similarity searches. In brief, iterative PSI-BLAST [44] (against UniProt database of 6 Apr, 2015, Number of letters: 172,526,934, Number of sequences: 461,263; E-value threshold of 1e-5), JackHMMER program from the HMMER3 package [36] (against: UniProtKB version 2014-06-17 and PDB version 2014-06-17; E-value threshold of 0.01), FFAS server [37] (against the regularly updated PDB, Pfam and SCOP databases) were used. MSAs of the various ferredoxin-like domains were generated using Profile Consistency Multiple Sequence Alignment (PCMA) [45] with default parameters.

As most families share a low sequence similarity, therefore, alignments generated by automated programs must be verified based on the actual structural data. Thus, a structure based manual correction of the MSAs was performed. This involved manual comparison of the automatically aligned regions and the actual structurally superimposing regions, verifying the accuracy of the MSA and manually correcting it if required. Particular care was taken to align the residues involved in forming conserved interactions including hydrogen bonding and interactions with heme moiety, and looking for subtle topological and structural features such as bulges, kinks, etc. that may help rationalize a distant evolutionary relationship [46].

### Structure-based methods

Dali [47] and TopSearch [48] tools were used to evaluate the structural similarity of the complete barrels and the individual ferredoxin-like domains with other structures in the PDB. Dali evaluates protein structural similarity and provides a Z-score as a measure of statistical significance based on intermolecular distance matrices [47]. TopSearch uses the TopMatch algorithm [49] to assess structural similarities among proteins while accounting for circular permutations and similarities in biological assemblies and asymmetric units. DaliLite program [50] was used for evaluating pairwise similarity among various structural domains. All protein structures were visualized and compared in the molecular visualization program PyMOL. Manual structural superimposition of structures was performed by defining the equivalent regions using the pair fitting command of PyMOL.

The topology diagrams of the ferredoxin α + β barrels for various protein families were sketched manually by referring to the three-dimensional structures.

### Ethics approval and consent to participate

Not Applicable.

### Consent for publication

Not Applicable.

### Availability of data and material

All the structural data used for this study is freely available from the Protein Data Bank (http://www.rcsb.org/pdb/home/home.do) and the sequences from the UniProtKB resource (http://www.uniprot.org/).

## Additional files

Additional file 1: Figure S1. Residue conservation between the heme-binding region of IsdG-like, OxdA, and Cld/Dyp/EfeB/HemQ. Ribbon diagrams of IsdG-like ferredoxin-like monomer (PDB identifier 2ZDP_A; colored purple), IsdG homolog without heme (PDB identifier 1TZ0_B; colored cyan), C-terminal heme-binding domain of OxdA-like (PDB identifier 3A16_A; colored red) and C-terminal heme-binding domain of Cld/DyP/EfeB/HemQ-like (PDB identifier 3NN1_A; colored green). Protein structures are shown as a trace of backbone C_α_ atoms and side chains of conserved residues around the heme moiety are as sticks. His299 (axial histidine residue) of 3A16_A is at an equivalent spatial position to Phe72 of 2ZDP_A, His75 of 1TZ0_B and His160 of 3NN1_A. Apart from the conserved axial histidine, Trp283, Trp292, His320 and Val322 of 3A16_A are also at the similar spatial position as Trp60, Trp69, His96 and Val98 of 1TZ0_A. Trp66 and Phe11 of 1TZ0_B structurally align with the Trp145 and Phe190 of 3NN1_A. The disordered region in the IsdG homolog without heme is shown as a dashed-connector. (TIF 54245 kb) Additional file 2: Figure S2. Flowchart of the methodology followed in this study. (TIF 40368 kb) Additional file 3: Ferredoxin α + β barrel structures in PDB (current till 30-12-15). (DOC 57 kb)

## Abbreviations

ABM: antibiotic biosynthesis monooxygenases
CDE: Cld DyP EfeB
Cld: chlorite dismutase
DyP: dye-decolorizing peroxidase
FFAS: fold and function assignment system
HTH: helix-turn-helix
MSA: multiple sequence alignment
OxdA: aldoxime dehydratase
PCMA: Profile Consistency Multiple Sequence Alignment
PDB: protein data bank
PSI-BLAST: position-specific iterative basic local alignment search tool
SCOP: structural classification of proteins
