# Supplementary figures and images for: Evolutionary relationships between heme-binding ferredoxin α + β barrels
Source: BMC Bioinformatics. 2016 Apr 18;17:168. doi: 10.1186/s12859-016-1033-6 (PMC4835899; doi:10.1186/s12859-016-1033-6)

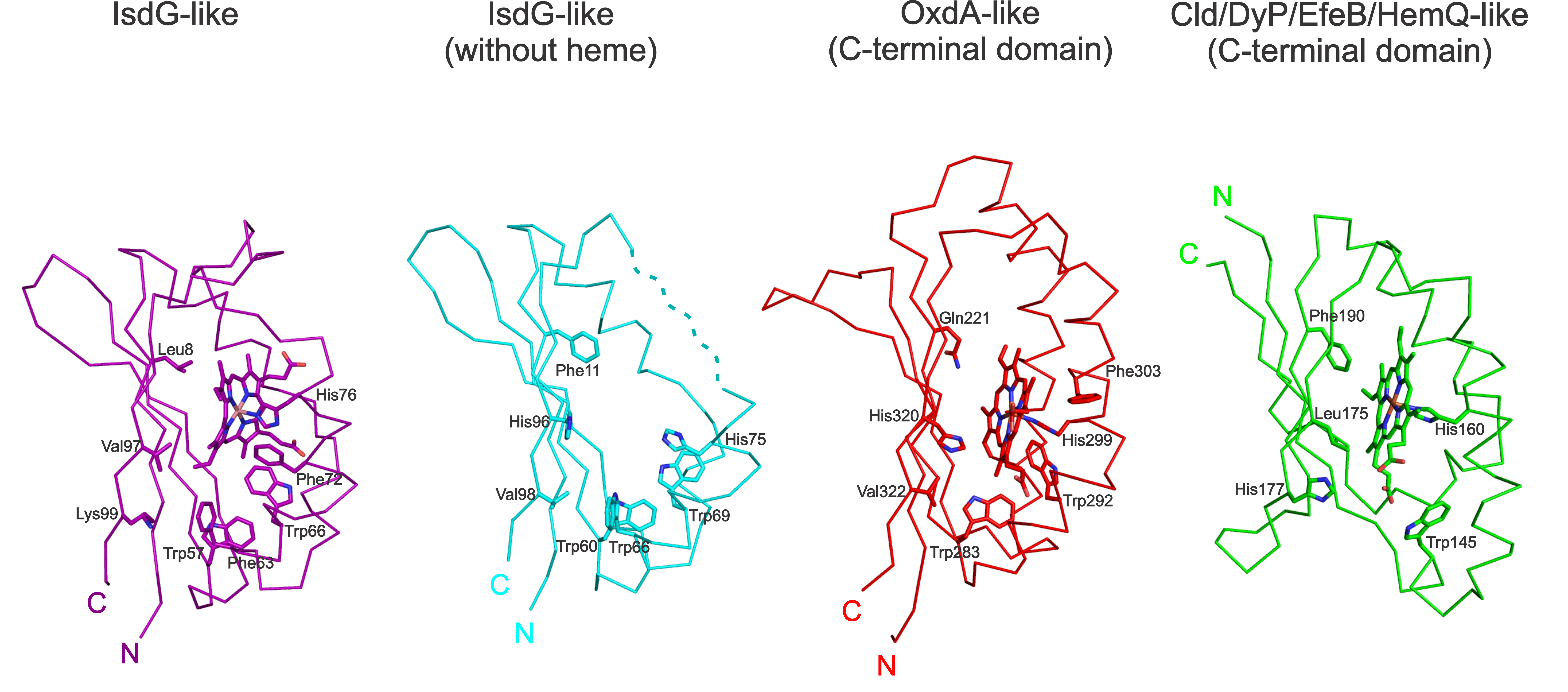

Supplement: Additional file 1: Figure S1. — Residue conservation between the heme-binding region of IsdG-like, OxdA, and Cld/Dyp/EfeB/HemQ. Ribbon diagrams of IsdG-like ferredoxin-like monomer (PDB identifier 2ZDP_A; colored purple), IsdG homolog without heme (PDB identifier 1TZ0_B; colored cyan), C-terminal heme-binding domain of OxdA-like (PDB identifier 3A16_A; colored red) and C-terminal heme-binding domain of Cld/DyP/EfeB/HemQ-like (PDB identifier 3NN1_A; colored green). Protein structures are shown as a trace of backbone Cα atoms and side chains of conserved residues around the heme moiety are as sticks. His299 (axial histidine residue) of 3A16_A is at an equivalent spatial position to Phe72 of 2ZDP_A, His75 of 1TZ0_B and His160 of 3NN1_A. Apart from the conserved axial histidine, Trp283, Trp292, His320 and Val322 of 3A16_A are also at the similar spatial position as Trp60, Trp69, His96 and Val98 of 1TZ0_A. Trp66 and Phe11 of 1TZ0_B structurally align with the Trp145 and Phe190 of 3NN1_A. The disordered region in the IsdG homolog without heme is shown as a dashed-connector. (TIF 54245 kb) [file 12859_2016_1033_MOESM1_ESM.tif]

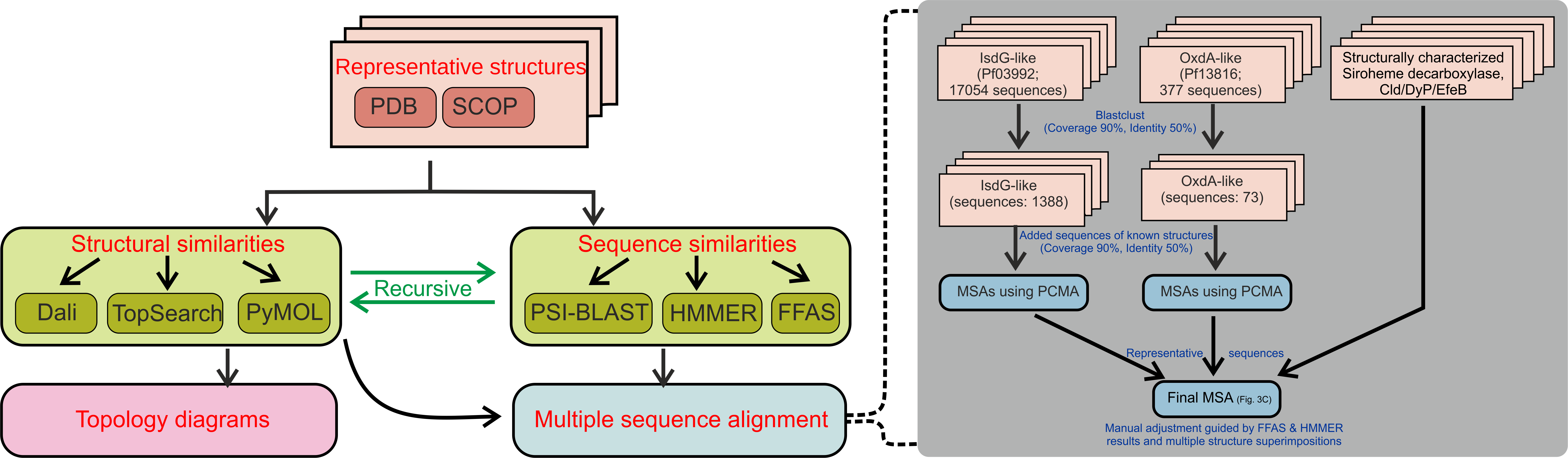

Supplement: Additional file 2: Figure S2. — Flowchart of the methodology followed in this study. (TIF 40368 kb) [file 12859_2016_1033_MOESM2_ESM.tif]
