# Supplementary material for: Evolutionary relationships between heme-binding ferredoxin α + β barrels
Source: BMC Bioinformatics. 2016 Apr 18;17:168. doi: 10.1186/s12859-016-1033-6 (PMC4835899; doi:10.1186/s12859-016-1033-6)
Supplement: Additional file 3: — Ferredoxin α + β barrel structures in PDB (current till 30-12-15). (DOC 57 kb) [file 12859_2016_1033_MOESM3_ESM.doc]

**Evolutionary relationships between heme-binding ferredoxin α+β barrels**

Giriraj Acharya, Gurmeet Kaur, Srikrishna Subramanian

**Supplementary File 1: ferredoxin α+β barrel structures in PDB (till 30-12-15)**

| **S.No.** | **Families** | **SCOPe v2.05**  **Family identifier** | **Pfam v29.0**  **identifier** | **PDB identifier(s)** |
| --- | --- | --- | --- | --- |
| 1 | Muconalactone isomerase, MLI | 54910 | PF02426 | 1mli,3znu,3znj,4fpi,3zo7 |
| 2 | Lrp/AsnC-like transcriptional regulator C-terminal domain | 69733 | PF01037 | 2cyy,1i1g,1ri7,2zny,2znz,2e1c,2cfx,2cg4,2p5v,2p6s,2p6t,2efn,2yx4,2yx7,2efo,2efp,2efq,2pn6,2e7x,2e7w,2pmh,4pcq,2cvi,,2zbc,2z4p,2w25,2qz8,2vbw,3i4p,2djw,2e1a,2ivm,2vc0,2w24,2vbx,2vc1,2vby,2vbz,2gqq,2w29,2dbb,2ia0,4ch7,4czc,4czd,4un1 |
| 3 | Actinorhodin biosynthesis monooxygenase ActVa-Orf6 | 82666 | PF03992 | 1lq9,1sqe,2zdp,3lgn,1n5s,3kg0,1n5q,1x7v,2zdo,3qgp,4fni,1tz0,3lgm,1n5t,1xbw,4fnh,1r6y,1n5v,3kg1,1tuv,2go8,1iuj,2ril,1y0h,2gff,3hx9,3qmq,2omo,4nl5,2bbe,4dpo,1q8b,4dn9,2fb0,3bm7,3e8o,3tvz,3mcs,3fj2,3fgv,3f44,3kng,3kkf,4fvc,4hl9,4jou,4npo,4oz5,4kia,4ae5,3fez,3gz7,2pd1,4zos |
|  | PG130-like | 102959 |  |  |
|  | Hypothetical protein YjcS | 102962 |  |  |
|  | PA3566-like | 110970 |  |  |
| 4 | Plant stress-induced protein | 89927 | PF07876 | 1tr0,1si9,1rjj,1q4r,1q53,2q3p,3fmb,3bgu,2qyc,3bn7,3bde,3bb5 |
| 5 | YciI-like | 102965 | PF03795 | 1mwq,1s7i,4lbh,4lbp,4lbi |
|  | DGPF domain (Pfam 04946) | 110962 |  |  |
| 6 | Polyketide synthesis cyclase | 110959 | PF04673 | 1tuw |
| 7 | Chlorite dismutase-like | 110965 | PF06778 | 1t0t,1vdh,4wws,3dtz,3nn1,3nn2,3qpi,4m05,4m09,4m07,3nn3,4m06,3nn4,4m08,3q09,2vxh,3q08,5a12,5a13 |
| 8 | Hypothetical protein YdhR | 117940 | PF08803 | 2hiq,1wd6,2asy,4za1 |
| 9 | NIPSNAP | 117943 | PF07978 | 1vqy,2ap6,1vqs |
| 10 | Dyp-type peroxidase-like | 143265 | PF04261 | 2gvk,2iiz,2d3q,2hag,4gu7,3vxi,3vxj,3afv,3mm1,3mm2,3mm3,4w7k,4w7l,4w7m,4w7o,3qns,4w7n,4w7j,4au9,4uzi,4hov,3qnr,3veg,3vee,3ved,3vec,3vef,2y4d,2y4e,2y4f,4gs1,4gt2,4grc,3o72,4g2c,5de0,5c2i |
| 11 | EthD-like | 143272 | PF07110 | 2ftr,3bf4 |
| 12 | Atu0297-like | 143275 | PF07045 | 2fiu,3lo3,3hhl,3dca |
| 13 | SOR-like | 143278 | PF07682 | 2cb2,2yav,2yax,2yaw,3bxv,3gn6 |
| 14 | YbaA-like | 160289 | PF07237 | 2okq |
| 15 | MmlI-like | 160292 | PF09448 | 2ifx,3hf5,3hds,3hfk |
| 16 | YiiL-like | 160298 | PF05336 | 1x8d,2qlw,2qlx |
| 17 | Aldoxime dehydratase* |  | PF13816 | 3a15,3a16,3a17,3a18,3w08 |
| 18 | DUF4242/SCO4226* |  | PF14026 | 4oi6,4oi3 |
| 19 | HapK* |  | PF11639 | 2jdj |
| 20 | Marine metagenome family DABB2 | 160295 |  | 2od4 |
| 21 | Marine metagenome family DABB1 | 160301 |  | 2od6,2op5 |
| 22 | Marine metagenome family DABB3 | 160306 |  | 2pgc |
| 23 | Chalcone isomerse* |  |  | 3zph,4c9s,4c9t,4d06 |

Total = 222

* Families not yet classified by SCOPe v2.05

PDBids in black- Classified by SCOPe v2.05

PDBids in purple- Classified by SCOPe v2.05 but not by Pfam v29.0

PDBids in green- Classified by Pfam v29.0 but not by SCOPe v2.05

PDBids in Red- Not yet classified by SCOPe v2.05 and Pfam v29.0
